# Editorial: HTLV-1 and EBV-related disorders: pathogenesis and clinical advances

**DOI:** 10.3389/fmed.2025.1742340

**Published:** 2025-12-01

**Authors:** Denis Miyashiro, Jorge Casseb, Makoto Sugaya, Hiroshi Yasui, José Antonio Sanches

**Affiliations:** 1 Department of Dermatology, University of São Paulo Medical School, São Paulo, Brazil; 2 International University of Health and Welfare, Narita, Japan; 3 Department of Hematology and Oncology, St. Marianna University School of Medicine, Kawasaki, Japan; 4 The Institute of Medical Science, The University of Tokyo, Tokyo, Japan

**Keywords:** HTLV-1, Epstein–Barr Virus (EBV), pathogenesis, clinical analysis, virus

Chronic infections caused by Human T-lymphotropic Virus type 1 (HTLV-1) and Epstein–Barr Virus (EBV) are characterized by profound immune dysregulation that predisposes individuals to infectious, inflammatory, and malignant disorders affecting multiple organs and systems. Both viruses have a complex interaction with the host immune response, leading to a wide clinical spectrum ranging from asymptomatic infection to life-threatening diseases.

HTLV-1, a deltaretrovirus with tropism for CD4^+^ T-cells, is endemic in Japan, the Caribbean, sub-Saharan Africa, South America, Australo-Melanesia, and focal regions of the Middle East (1). The virus induces CD4^+^ T-cell dysfunction, predisposing to severe opportunistic infections (such as disseminated strongyloidiasis), inflammatory conditions (including infective dermatitis, HTLV-1–associated myelopathy/tropical spastic paraparesis [HAM/TSP], and HTLV-1–associated uveitis), and adult T-cell leukemia/lymphoma (ATLL), an aggressive lymphoproliferative malignancy (2).

EBV, a herpesvirus with global distribution, primarily infects B cells but can also affect T and NK cells. After initial infection of epithelial cells, the virus establishes latency as circular plasmids in memory B cells. Immunological responses, modulated by host HLA polymorphisms, largely determine the outcome of infection. Primary EBV infection is often asymptomatic in early childhood, whereas infection during adolescence or adulthood can present as infectious mononucleosis, characterized by an exaggerated CD8^+^ T-cell response. Persistent EBV infection is associated with a spectrum of diseases, including lymphoproliferative disorders (e.g., Burkitt's lymphoma, Hodgkin's disease, extranodal NK/T-cell lymphoma, post-transplant lymphoproliferative disease), nasopharyngeal carcinoma, hydroa vacciniforme, and chronic active EBV disease (3). The higher prevalence of some EBV-related disorders in Asian and Latin American populations suggests underlying genetic susceptibility (4, 5).

## EBV-related studies

Pereira et al. analyzed HLA patterns and immune responses in EBV-infected patients, stratifying participants into three groups: those with primary infection (IgM^+^/IgG^−^), those undergoing serological class switching (IgM^+^/IgG^+^), and those with past infection (IgM^−^/IgG^+^). The study demonstrated significant differences in inflammatory cytokine profiles and HLA class distributions among these groups, suggesting that the serological response to EBV is influenced by HLA background. Notably, HIV co-infection acted as an additional determinant of immune modulation. The association between the HLA class II DRB1^*^09 allele and primary EBV infection supports its potential role in systemic inflammation and immune regulation, particularly in HIV-infected individuals.

Complementing these findings, Rostgaard et al. investigated the risk of infectious mononucleosis in a Danish population using a large-scale national database. The authors explored whether previous antimicrobial use or exposure periods during adolescence could modulate susceptibility to infectious mononucleosis by evaluating sibling pairs. Although these associations were not confirmed, the study provides valuable epidemiological insight into potential environmental and immunological modifiers of EBV infection.

Together, these studies expand the current understanding of EBV pathogenesis, emphasizing how host genetic factors (e.g., HLA subtypes), co-infections (e.g., HIV), and prior immune exposures can shape clinical outcomes.

## HTLV-1-related studies

Carneiro et al. conducted the first Brazilian study evaluating HTLV-1 prevalence among transgender women—a recognized high-risk population due to higher prevalence of injection drug use and unprotected sexual practices. In a cross-sectional cohort of 235 transgender individuals, three cases of HTLV-1 infection were identified (1.3%). Although prevalence was low, the study highlights the urgent need for targeted health programs and preventive interventions to mitigate HTLV-1 transmission and associated comorbidities in vulnerable populations.

Fernandes et al. investigated small RNA (sRNA) expression profiles in HTLV-1, HTLV-2, and healthy controls in Brazil, exploring their potential diagnostic, prognostic, and therapeutic applications. HTLV-2, endemic among Amerindian and African Pygmy populations, has been associated with HAM/TSP and increased infection susceptibility. The study revealed distinct sRNA expression patterns between HTLV-1 and HTLV-2 infected individuals, suggesting that specific sRNAs may serve as novel biomarkers and therapeutic targets for HTLV-related diseases.

In a longitudinal cohort spanning over three decades (1991–2024), Rosadas et al. analyzed the incidence of HAM/TSP in the United Kingdom—a non-endemic region for HTLV-1. The authors observed an incidence rate of 1.35% among HTLV-1 positive individuals, consistent with data from Brazil and the Caribbean. Most cases originated from Caribbean and West African immigrants, underscoring the role of migration in HTLV-1 dissemination. Patients with HAM/TSP had significantly higher proviral loads, increased mortality, and markedly reduced quality of life—ranking among the lowest scores compared with over 130 chronic conditions. These findings reinforce the importance of clinical suspicion for HTLV-1–associated neurological diseases, even in non-endemic regions.

Valente et al. described a large cohort of ATLL patients in Brazil, focusing on the diverse cutaneous manifestations. Among 44 patients, in addition to the well-described ATLL specific skin lesions (patches, plaques, papules, nodules/tumors, erythroderma, and purpura), other less frequent manifestations were observed, including palmoplantar hyperkeratosis, panniculitic nodules, cutis laxa, and periocular heliotrope-like lesions. A detailed description of histopathological findings, laboratory results, and survival data was provided. Complementarily, Lewitt and Gru presented a comprehensive review of ATLL-related skin lesions, underscoring the diagnostic importance of recognizing these dermatological features in the context of HTLV-1 infection.

Finally, Shegefti et al. presented a detailed review of the immune mechanisms underlying HTLV-1–associated inflammation across multiple clinical contexts, including HAM/TSP, ATLL, and other inflammatory syndromes. The review synthesizes recent progress in understanding how viral persistence drives chronic immune activation and tissue damage.

## Conclusion

The eight manuscripts comprising this Research Topic, “*HTLV-1 and EBV-related Disorders: Pathogenesis and Clinical Advances,”* collectively provide valuable insights into the epidemiology, immunopathogenesis, and clinical spectrum of these persistent viral infections. The contributions deepen our understanding of host–virus interactions, genetic and immunologic determinants of disease progression, and potential diagnostic and therapeutic approaches.

Despite significant advances, many aspects of HTLV-1 and EBV pathogenesis remain unresolved. Continued multidisciplinary research integrating virology, immunology, genomics, and clinical science is essential to elucidate mechanisms of viral persistence, immune evasion, and malignant transformation. Through these studies, the goals of this Research Topic have been achieved, advancing our comprehension of infections that range from silent carriage to severe, life-threatening malignancies with profound impact on global health and quality of life.

## References

[1] Gessain A Cassar O. Epidemiological aspects and world distribution of HTLV-1 infection. Front Microbiol. (2012) 3:388. doi: 10.3389/fmicb.2012.00388 23162541 PMC3498738

[2] Miyashiro D Sanches JA. Cutaneous manifestations of adult T-cell leukemia/lymphoma. Semin Diagn Pathol. (2019) 37, 81–91. doi: 10.1053/j.semdp.2019.07.010 31387753

[3] Quintanilla-Martinez L Swerdlow SH Tousseyn T Barrionuevo C Nakamura S Jaffe ES. New concepts in EBV-associated B, T, and NK cell lymphoproliferative disorders. Virchows Archiv. (2022) 482:227–44. doi: 10.1007/s00428-022-03414-4 36216980 PMC9852222

[4] Plaza JA Gru AA Sangueza OP Lourenco SV Puccio FB Sanches JA . An update on viral-induced cutaneous lymphoproliferative disorders. J Am Acad Dermatol. (2023) 88:965–80. doi: 10.1016/j.jaad.2021.11.068 36041557

[5] Gru AA Plaza JA Sanches JA Miyashiro D Sangueza OP Puccio FB . An update on Epstein-Barr virus-and human T-lymphotropic virus type-1-induced cutaneous manifestations. CME Part II J Am Acad Dermatol. (2023) 88:983–98. doi: 10.1016/j.jaad.2022.07.063 36049582

